# Quality of life and psychological functioning in postmenopausal women undergoing aromatase inhibitor treatment for early breast cancer

**DOI:** 10.1371/journal.pone.0230681

**Published:** 2020-03-26

**Authors:** Gabriella Martino, Antonino Catalano, Rita Maria Agostino, Federica Bellone, Nunziata Morabito, Carmen Giulia Lasco, Carmelo Mario Vicario, Peter Schwarz, Ulla Feldt-Rasmussen

**Affiliations:** 1 Department of Clinical and Experimental Medicine, University Hospital of Messina, Messina, Italy; 2 Unit of Medical Oncology, Grand Metropolitan Hospital Bianchi Melacrino Morelli, Reggio-Calabria, Italy; 3 Department of Cognitive Sciences, Psychology, Education and Cultural Studies, University of Messina, Messina, Italy; 4 Department of Medical Endocrinology, Copenhagen University Hospital, Rigshospitalet, Copenhagen, Denmark; State University of New York Upstate Medical University, UNITED STATES

## Abstract

**Introduction:**

Aromatase inhibitors (AIs) dramatically increased breast cancer (BC) survival, leading to enhanced attention to their long-term consequences on psychological functioning. Conflicting data has been examined regarding the association between AIs administration and the clinical psychological features in BC survivors (BCSs).

**Purpose:**

As psychological symptoms often occur in such chronic diseases, our study aimed at exploring anxious and depressive symptoms and the perceived quality of life (QoL) in BCSs assessed for osteoporosis.

**Methods:**

The total sample consisted of a clinical sample of 51 outpatient postmenopausal women, diagnosed with BC, and a control group composed of 51 healthy postmenopausal women. All recruited participants were evaluated through the clinical gold standard interview and completed the following self-rating scales: the Hamilton Anxiety Rating Scale, Beck Depression Inventory II edition, and 36-Item Short Form Health Survey, which were administered at baseline and after 6 months in BCSs in AIs treatment, compared with controls. Moreover, all participants were assessed for vitamin D status, bone mineral density (BMD) and subclinical vertebral fractures. Data regarding age, age at menopause, body mass index (BMI), smoking habits and alcohol consumption was collected.

**Results:**

BCSs (n = 51) showed higher anxious and depressive symptoms, and lower perceived QoL vs. controls (n = 51) (p<0.05 for all). After 6 months of treatment with AIs, BCSs showed significant reduction of anxious and depressive symptoms and a significantly higher perceived QoL for both physical and mental components, vs. controls.

**Conclusions:**

The improvement of clinical psychological features and perceived QoL was associated with AIs treatment in women being treated with, for early breast cancer. Further studies are needed to obtain a deeper comprehension of the correlation between clinical psychological and physical features in BCSs.

## Introduction

One of the major health diseases affecting women worldwide is breast cancer (BC), which is the most prevalent cancer and the first cause of cancer mortality among women, although in these last decades, a significant reduction in BC mortality due to improved screening programs and treatments has been observed [1]. Recently, there has been an increased interest in the impact of BC and its treatments on psychological functioning and the perceived quality of life (QoL) [2–4].

Most BC survivors (BCs) are estrogen receptor positive inducing advantageous outcomes by adjuvant endocrine therapy (ET) [5]. It is known that the aromatase enzyme converts androgens into estrogens and represents the main source of peripheral estrogen production in postmenopausal women. Aromatase inhibitors (AIs), blocking endogenous estrogen synthesis through the inhibition of peripheral aromatase, represent the gold standard adjuvant hormone therapy for postmenopausal women with hormone receptor-positive BC. AIs treatment has been associated with adverse events such as increased bone loss, musculoskeletal pain, impaired lipid profile and cardiovascular risk, but also with mood disturbances, anxiety and memory deficit [6–8]. Physical and psychological side effects seriously impair women’s psychological balance and perceived QoL and may negatively influence the participation in medical care and adherence to every fundamental prescription [9–17]. In fact, several studies demonstrate the importance played by traumatic factors both on mental health and mood which could also lead to an increased suicidal risk and cognitive decline [18–24]. A recent evidence demonstrates the role of motivation and its relationship with anxiety, depression and QoL in subjects with chronic diseases [25–30].

Several studies examined the impact of ET on cognitive functioning in BC survivors (BCSs) detected at different times from diagnosis and according to various treatments and duration. Some evidence suggested that hormonal changes during specific treatments do not provoke cognitive decline in patients BCSs in the first years from diagnosis [31]. The occurrence of severe perceived cognitive deficits have been noted, above all in attention and memory, and worse QoL in BCSs who were undergoing adjuvant therapy, which are disruptive for BCSs in their work life because of lack of performance [32,33].

Previous studies have highlighted the physical adverse effects in BCSs being treated with AIs, focusing on emotional distress [10, 34,35].

Bidstrup et al. observed that a young age, not having a partner, less education, and receiving chemotherapy but not radiotherapy might identify BCSs whose psychological distress lasted eight months after BC diagnosis [36]. Vance et al. reported also that symptoms of physical and psychological distress may be associated with weight change after treatment [37].

Nevertheless, personality and physical complaints resulting from adjuvant treatment distinguished different distress trajectories [38].

High patient-perceived burden from physical symptoms, and high coping self-efficacy suggest a transient, self-limiting distress trajectory, while patients experiencing chronic distress, and those developing distress following treatment completion only cannot be identified by a single initial assessment. [39–42].

Ho et al. underlined the key role of timely recognition of anxiety and depression, during the treatment and survivorship phases of BC trajectory [43].

Takei et al. studied psychological distress, and adverse events in BCSs who received ETs, finding that HRQoL was better in BCSs treated with tamoxifen than those treated with exemestane or anastrozole [44]. Moreover, Donovan et al. suggested a large prevalence of persistent depressive symptoms at the start of adjuvant treatment, focusing the relevance of psychological screening during the therapy [45].

Conversely, Schilder et al. [46] detected depressive symptoms in BCSs treated with ET and found no significant differences in comparison with healthy controls.

In BCSs, AIs physical adverse effects (e.g. hot flushes, palpitation, bone or joint pain, muscle stiffness) are commonly reported as well as psychological effects (e.g. anxiety and depressive symptoms) [10].

As many BCSs perceive a range of symptoms as a consequence of ET, Rosenberg et al. (2015) suggested attention to these symptoms may improve adherence and QoL, optimizing survival [10].

Ates et al. (2016) described the psychosocial and medical characteristics of BCSs initiating ET and evaluated emotional distress according to their psychosocial and medical characteristics, highlighting that these patients’ features were related to emotional distress[34]. Schilder et al. (2009) detected depressive symptoms in BCSs treated with different ET and found no significant differences in comparison with the healthy group[46]. Differently, Maas et al. (2015) found a higher prevalence of depressive symptoms among BCSs than in the general female population, while they didn’t find an increased prevalence of anxiety[35].

It is well known that emotional distress is reported in postmenopausal women who have a greater risk of developing both BC and osteoporosis[47].

On the basis of this data we aimed at exploring emotional distress, in an Italian sample of postmenopausal BCSs assessed for osteoporosis, focusing on anxiety levels, depressive symptoms and health related QoL before starting therapy and 6 months after initiation of AIs treatment.

## Materials and methods

### Participants

We recruited a group of postmenopausal women with a diagnosis of BC and a group of healthy controls. Both groups were referred to the Outpatients Clinics at the Department of Clinical and Experimental Medicine, University Hospital of Messina, Italy, for BMD evaluation by DXA-scanning. Research eligibility criteria included: postmenopausal age, graduation from primary school or higher; newly diagnosed early BC staged 0, I, II, or IIIA; non-metastatic hormone receptor positive BC; completed surgical treatment; concluded chemotherapy and radiation therapy when prescribed. All treatments ended 3 months before the start of the study.

Exclusion criteria were: known neurological or psychiatric diseases, according to the Diagnostic and Statistical Manual of Mental Disorders (DSM-5) criteria [48] which could interfere with the study; previous bone fractures; previous cancer; autoimmune and endocrine diseases; cardiovascular, respiratory, liver or kidney failures; psychopharmacological therapy and use of steroid, hormone treatment or any active bone agents; already started adjuvant aromatase inhibitor administration.

### Ethics statement

The study was approved by the Institutional Ethical Committee of the University Hospital “Gaetano Martino”, University of Messina, Italy. The research was conducted with respect for the rights of all participants and data was analysed entirely anonymously. Participants were evaluated by researchers in Clinical Psychology in collaboration with physicians. All subjects were thoroughly informed about the research aim of the study and gave written informed consent in accordance with the Declaration of Helsinki [49] and its subsequent revisions. All intervention, including rating scales administration and physical parameters detection were performed as a part of daily clinical assessment of patients.

### Measures

#### Demographical and medical data

Data on each participant data regarding age, age at menopause, smoking habits, alcohol consumption and BMI was collected. Medical information comprised data on vitamin D status, BMI, BMD and data on subclinical vertebral fractures.

#### Clinical psychological evaluation

A gold standard interview to detect patient’s mental status was performed by a researcher in clinical psychology in a confidential setting [50–52]. This gold standard interview was complementary combined with the psychodiagnostic administration of the following self-report scales and questionnaires: Beck Depression Inventory Second Edition (BDI-II), Hamilton Anxiety Rating Scale (HAM-A), the Italian version of Short Form-36 (SF-36) questionnaire. Particularly, BDI-II, consisting of 21 items, was administered to detect the presence and severity of depressive symptoms, based on a range from 0 to 63, with higher scores reflecting more severe symptoms [53]. In the present study the reliability (Cronbach’s α) for the total score was .89.

HAM-A comprising 14 items, was used to detect anxiety levels. Each item is scored from 0 to 4, depending on the severity of perceived anxiety. It measures both psychological and somatic anxiety. In the area of psychic anxiety it measures anxious moods, tension, fears, insomnia, intellectual and depressed mood. In the area of somatic symptoms it measures the sensory, cardiovascular, respiratory, gastrointestinal, genitourinary, autonomic and observed behaviour at the time of interview [54]. In the current study the reliability (Cronbach’s α) was .87 for the total score, and .83 and .77 for psychological and somatic anxiety respectively.

The Italian version of the SF-36 survey was administered to detect participants’ health perceived QoL [55,56] exploring the following eight dimensions: physical functioning, social functioning, role limitations because of physical problems, role limitations because of emotional problems, health, vitality, pain, and general health perception. Each dimension was scored from 0 to 100 points, with higher scores indicating lower limitations and better perceived QoL. Physical Component Summary (PCS) and Mental Component Summary (MCS) were also evaluated [57] to analyze both physical and mental well-being. In the present study the reliability (Cronbach’s α) was .83 and .82 for PCS and MCS respectively, with acceptable values for each dimension as follows: physical functioning (.85), role-physical (.78), bodily pain (.71), general health (.80), vitality (.79), social functioning (.72), role-emotional (.77), and mental health (.82).

#### Clinical characteristics

Physical evaluation was conducted measuring height and weight, according to standard procedures, and vitamin D status was assessed by HPLC, measuring 25(OH)D serum concentrations; BMD was measured at the lumbar spine (mean of L1-L4) in anteroposterior projection, and at femoral neck by dual-energy X-ray absorptiometry (DXA) (Hologic Discovery) [58]. A dorso-lumbar X-ray scan was applied in lateral projection, according to Genant’s classification, to exclude previous subclinical vertebral fractures [59].

BCSs and controls were compared at baseline and 6 months for all explored parameters, except for 25(OH)D and BMD.

### Statistical analysis

Statistical analysis was performed using the MedCalc software (version 10.2.0.0; Mariakerke, 173 Belgium). Comparisons between groups were performed by an unpaired t -test or Mann-Whitney test and within-group comparisons were determined by paired t- test or Wilcoxon matched paired rank sum test for paired data as appropriate. The χ2 test was performed to calculate differences in the proportion of categorical variables. Spearman’s coefficient was used to measure the degree of association between two variables. Multiple regression was performed to analyse the association between a dependent variable and one or more explanatory variables. Values of p ≤0.05 indicated statistical significance. All reported p values were two-sided.

## Results

102 postmenopausal women were recruited and completed the study according to the protocol. We screened 146 postmenopausal women, of whom 62 were diagnosed with BC and 84 were healthy controls. Due to inclusion and exclusion criteria we enrolled only 51 BCSs (82%) and 51 healthy controls (60%).

Before entering the study the BCSs had received surgical treatment (100%), chemotherapy (60%) and radiotherapy (90%), in accordance with routine oncological prescription. The 51 BCSs received daily AIs and bimonthly cholecalciferol 25,000 UI whereas the 51 controls solely received bimonthly cholecalciferol 25,000 UI.

The main clinical characteristics of the 102 participants at baseline are shown in Table 1.

**Table 1:** Baseline main clinical characteristics of all participants, breast cancer survivors (BCSs) and controls.

	Total *(n = 102)*	BCSs *(n = 51)*	Controls *(n = 51)*	p value
**Age** *(yr*.*)*	66.5±9.1	66.9±8.7	66±10.9	NS
**Age at menopause** *(yr*.*)*	47.3±5.4	47.±4.8	47.5±5.1	NS
**BMI** *(Kg/m*^*2*^*)*	24.4±4.9	24.5±5.5	24.4±4.2	NS
**Current smoking** *[n(%)]*	10 (10)	5 (10)	5 (10)	NS
**Alcohol ≥ 3units/day** *[n(%)]*	0	0	0	NS
**S-25(OH)D** *(ng/ml)*	26.9±10	25.1±7.4	28.7±11.7	NS
**Bone mineral density**				
**Lumbar spine T-score** *(SD)*	-2 ± 1	-2.1 ± 1	-1.9 ± 0.9	NS
**Femoral neck T-score** *(SD)*	-1.8 ± 0.6	-1.8 ± 0.6	-1.8 ± 0.7	NS
**Anxiety levels**				
**HAM-A score**	27.8±7.11	33.2±4.1	22.3±5*	<0.0001
**HAM-A somatic symptom score**	11.9±3.9	14.5±2.8	9.3±3.2	<0.0001
**HAM-A psychic symptom score**	15.9±3.8	18.7±2.4	13±2.9	<0.0001
**Depression severity**				
**BDI-II score**	7.2±3.1	8.6±2.6	5.9±3.1	<0.0001
**Perceived Quality of Life–SF-36**				
**Mental health**	28 (20 to 52)	28 (17 to 32)	44 (21 to 56)	0.001
**Role emotional**	0 (0 to 33)	0 (0 to 0)	33 (0 to 66)	<0.0001
**Social functioning**	50 (25 to 62)	37 (25 to 50)	50 (37 to 62)	0.0003
**Vitality**	35 (25 to 50)	30 (20 to 40)	40 (30 to 55)	0.0008
**General health**	40 (25 to 52)	30 (20 to 40)	45 (35 to 52)	0.0011
**Bodily pain**	41 (22 to 52)	30 (22 to 41)	41 (22 to 74)	0.004
**Role physical**	0 (0 to 50)	0 (0 to 0)	25 (0 to 75)	0.0001
**Physical functioning**	55 (30 to 75)	35 (20 to 55)	75 (55 to 90)	<0.0001

*Values are expressed as mean ± SD or median (IQR) as appropriate*. *BMI = Body Mass Index; S-25(OH)D = 25-hydrossi-vitamin D serum level; HAM-A = Hamilton Anxiety Rating Scale; BDI-II = Beck Depression Inventory II edition; SF-36 = Short Form Survey Instrument*.

At the baseline, we found no significant differences between the two groups regarding age, age at menopause, BMI, smoking habits, alcohol consumption, serum 25(OH)D concentration and BMD. The two groups showed significant differences at HAM-A, both in somatic and psychic scores. Mainly, BCSs obtained the highest HAM-A scores, reflecting higher anxiety levels in comparison with controls. The two groups also showed a significant difference at BDI-II, as the BCSs demonstrated higher scores, reflecting higher depressive symptoms in comparison with controls. Moreover, the two groups showed a significant difference at SF-36 scores for each of the eight explored domains. Particularly, there were lower scores for each domain in BCSs, in comparison with controls, reflecting BCSs’ worse perceived QoL.

Psychological features after 6 months are shown in comparison with the baseline in Table 2.

**Table 2:** Changes in psychological features in patients with breast cancer and controls at baseline and after 6 months of treatment with aromatase inhibitors.

	BC *(n = 51)*			Controls *(n = 51)*		
	Baseline	6 months		Baseline	6 months	
**Anxiety levels**			p value			p value
HAM-A score	33.2±4.1	30±3.7	<0.001	22.3±5	20±4.4	<0.001
HAM-A somatic symptom score	14.5±2.8	13.2±2.6	0.02	9.3±3.2	8.2±2.5	0.05
HAM-A psychic symptom score	18.7±2.4	17.2±2.2	0.002	13±2.9	11.9±2.8	0.04
**Depression severity**						
BDI-II score	8.6±2.6	8±2.6	0.001	5.9±3	5.6±2	NS
**Perceived quality of life**						
PCS	32.3±7.2	33.8±6.1	0.006	40.5±9.1	45.4±8	<0.001
MCS	27.9±5.4	34±6.2	<0.001	33.5±9.8	39.3±9.2	<0.001

*Data is reported as mean ± SD*. *BC = Breast Cancer; HAM-A = Hamilton Anxiety Rating Scale; BDI-II = Beck Depression Inventory II edition; PCS = Physical Component Summary; MCS = Mental Component Summary*.

Particularly, BCSs showed a significant difference of anxiety levels between the baseline and 6 month detections, with decreased anxious symptoms at the end of the study. Moreover, we noticed that at 6 months BCSs showed decreased depressive symptoms, even if it was not significantly different in comparison with baseline. Also BCSs at 6 months showed a statistically significant better perceived QoL in comparison with baseline. Additionally, controls showed a significant reduction of anxiety levels at 6 months in comparison with the baseline and they also presented decreased depressive symptoms, which were not significantly different from the baseline, as we observed instead in BCSs. Furthermore, controls had a significantly different perceived QoL, with higher scores at the end of the study.

**Fig 1:**
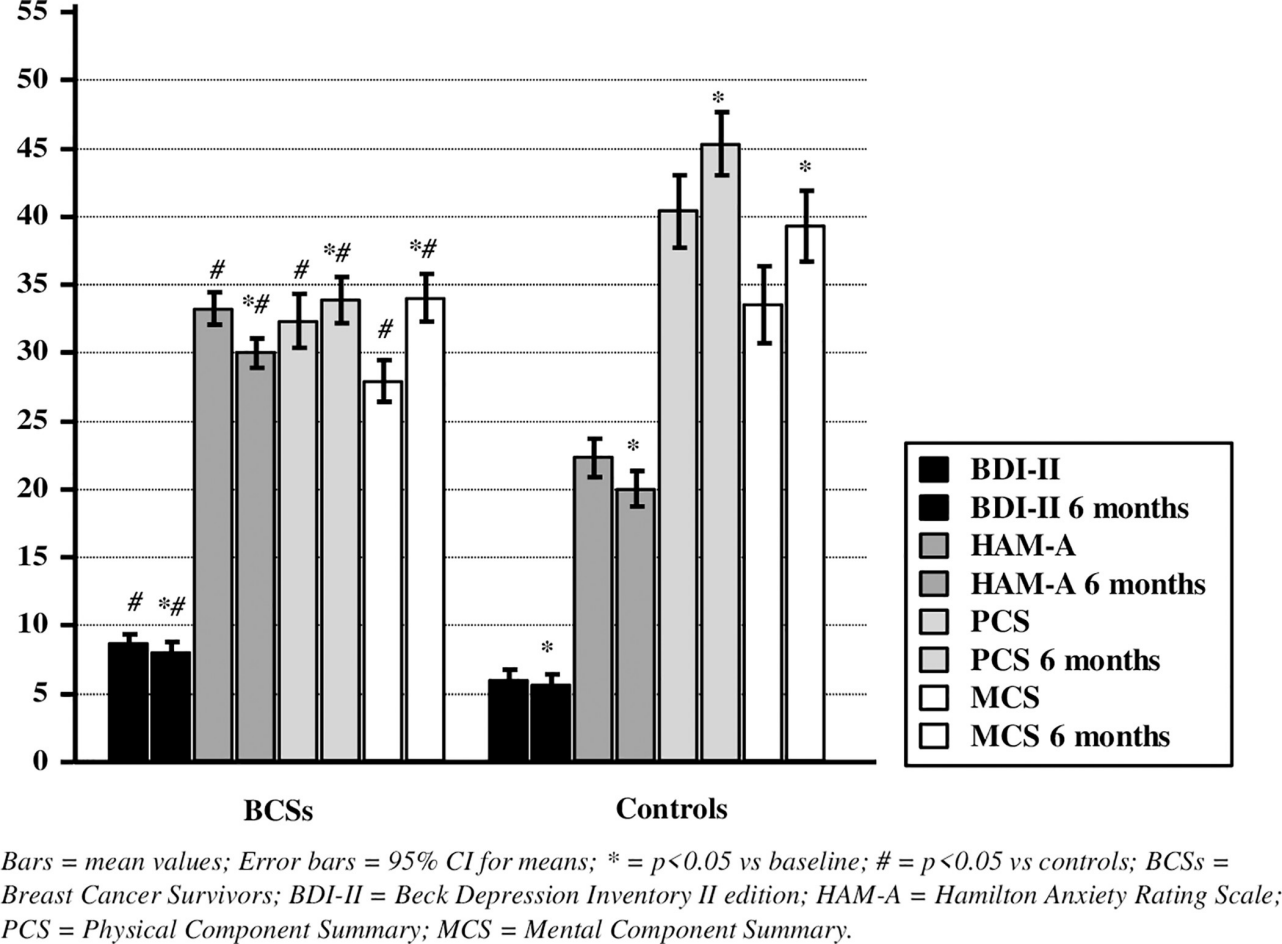
Clinical psychological features and comparison at baseline (*Left column of each signature*) and after 6 months (*Right column of each signature*). Bars = mean values; Error bars = 95% CI for means; * = p<0.05 vs baseline; # = p<0.05 vs controls; BCSs = Breast Cancer Survivors; BDI-II = Beck Depression Inventory II edition; HAM-A = Hamilton Anxiety Rating Scale; PCS = Physical Component Summary; MCS = Mental Component Summary.

The main outcome, at 6 months, consisted of higher anxious and depressive symptoms and lower perceived QoL in BCSs as compared to controls (Fig 1).

Besides, at baseline and after 6 months there were statistically significant differences in both somatic and psychic anxiety in both patients and controls (Table 2).

Anxiety levels, depressive symptoms and perceived QoL were significantly associated at baseline in all participants, and they were also related with age (Table 3).

**Table 3:** Correlation analysis at baseline between the studied variables of all participants.

	Age	Age at menopause	BMI	HAM-A score	BDI-II score	PCS	MCS
**Age**		r = 0.13; p = 0.19	r = 0.1; p = 0.28	r = 0.15; p = 0.12	**r = 0.25; p = 0.01**	**r = -0.52; p<0.001**	**r = -0.34; p<0.001**
**Age at menopause**	r = 0.13; p = 0.19		r = 0.11; p = 0.27	r = -0.03; p = 0.74	r = 0.07; p = 0.49	r = -0.14; p = 0.16	r = -0.14; p = 0.16
**BMI**	r = 0.1; p = 0.28	r = 0.11; p = 0.27		r = -0.12; p = 0.21	r = -0.15; p = 0.12	r = -0.08; p = 0.38	r = 0.10; p = 0.31
**HAM-A score**	r = 0.15; p = 0.12	r = -0.03; p = 0.74	r = -0.12; p = 0.21		**r = 0.63; p<0.001**	**r = -0.49; p<0.001**	**r = -0.53; p<0.001**
**BDI-II score**	**r = 0.25; p = 0.01**	r = 0.07; p = 0.49	r = -0.15; p = 0.12	**r = 0.63; p<0.001**		**r = -0.54; p<0.001**	**r = -0.46; p<0.001**
**PCS**	**r = -0.52; p<0.001**	r = -0.14; p = 0.16	r = -0.08; p = 0.38	**r = -0.49; p<0.001**	**r = -0.54; p<0.001**		**r = 0.39; p<0.001**
**MCS**	**r = -0.34; p<0.001**	r = -0.14; p = 0.16	r = 0.10; p = 0.31	**r = -0.53; p<0.001**	**r = -0.46; p<0.001**	**r = 0.39; p<0.001**	

*BMI = Body Mass Index; HAM-A = Hamilton Anxiety Rating Scale; BDI-II = Beck Depression Inventory II edition; PCS = Physical Component Summary; MCS = Mental Component Summary*. *Significant values (p<0*.*05) are reported in bold*.

At baseline, the 25(OH)D serum concentration was inversely related with both age (r = -0.33; p = 0.001) and with the HAM-A score (r = -0.19; p = 0.05), while it was positively related to PCS (r = 0.23; p = 0.01). Furthermore, in BCSs an association between the 25(OH)D concentration and Δ PCS (r = -0.33; p = 0.02), as well as between Δ HAM-A and Δ MCS (r = -0.29; p = 0.03) was found. Finally, we performed stepwise multiple regression analysis assuming Δ MCS (model 1), and Δ PCS (model 2), as dependent variables in two distinct models, including age, age at menopause, serum 25(OH)D, Δ HAM-A score and Δ BDI-II score as explanatory variables. The Δ HAM-A score was the only predictor of Δ MCS (β = 0.55, p = 0.03, SE = 0.25) (model 1), while patient’s age was the only predictor of Δ PCS (β = 0.70, p = 0.005, SE = 0.24) (model 2). However, in the control group there were no significant associations in Δ values for any explored psychological variable.

## Discussion

The main finding of this study is that BCSs showed decreased anxiety levels and depressive symptoms, presenting a better perceived QoL after 6 months of AIs treatment, compared with the baseline.

It is known that during the so-colled “re-entry” period, BCSs are usually distressed about the alteration of former roles, the decline in interpersonal support, and the lingering physical and psychological effects of diagnosis and treatment. This is in agreement with our findings that highlight a worse psychological state at the baseline evaluation, after completed surgical treatment and concluded chemotherapy and/or radiation therapy [60].

At the end of curative therapy, BCSs often receive long-term prophylactic AIs treatment to reduce BC relapses [6,61–63]. However, the treatment may be burdened by clinical psychological impairment due to estrogenic deprivation and their consequences on the brain [2–4].

The estrogenic actions on brain tissue and functioning has been intensively studied, including its activity on brain receptors, located in the hippocampus and cerebral cortex. Estrogen may have positive effects on neurotransmitters involved in cognitive processes and may also have a protective role against ischemic brain injuries through its anti-inflammatory action which enhances cell survival, improving blood flow and glucose transport in the brain [64–66]. There is also evidence suggesting estrogen could increase the risk of stroke and dementia [67,68]. However, there is conflicting data on the effects of both estrogen replacement and deprivation on cognitive function in clinical settings [68–71]. Bender et al. [72–77] highlighted decreased cognitive functioning in BCSs prior to initiation ET that did not appear to be influenced by treatment. Particularly, comparing anxiety levels between groups and exploring variation over time, they observed that women were more anxious at the baseline, while they were less anxious at 6 months. They found neither depressive symptoms nor fatigue were consistently associated with the cognitive function factors.

Aromatase inhibitor treatment, inducing estrogenic suppression, could provoke several adverse effects among which the aromatase inhibitor-associated musculoskeletal syndrome could adversely affect the health-related QoL of breast cancer survivors [8]. Conversely, in a recent multicenter study Taira et al. [78] suggested that neo adjuvant AIs induced a significant improvement of depressive and anxious symptoms and a better perceived QoL in a four month observation period in BCSs before surgical treatment.

In a recent cross-sectional descriptive and correlational study focused on psychosocial characteristics, Ates et al. [34] investigated the relationship between psychological and medical characteristics and self-reported emotional distress in BCSs who were treated with ET. Particularly they found that emotional distress was relatively higher among patients in the first two years of treatment, without any significant statistical difference in comparison with the following three years of treatment. To evaluate emotional distress, Ates and colleagues [34] administered the hospital anxiety and depression scale (HADs), a self-reported scale validated to detect the eventual presence of both anxiety and depressive states [79]. HADs is usually administered in clinical oncology and psychology services, but it does not allow clinicians to constructively discriminate between anxious and depressive symptoms, while it is more functional to measure the global entity of emotional distress [80,81].

Aromatase inhibitors may increase bone loss of up to 13% the first year of treatment and increase the risk of osteoporotic fractures risk as compared to healthy controls [58]. Bone fractures are by themselves associated with a higher risk of morbidity and mortality. The preventable increased risk of fractures due to the effects of AIs treatment in BCSs represents another source of worry which could further compromise their perceived QoL.

Maas et al. showed in their systematic literature review that the results on depression scales suggested an increase in risk of symptoms of depression, varying from 9.4% to 66%, in BCSs one year after the diagnosis, which then decreased over the following years. The prevalence of anxiety ranged from 17.9% to 33.3% [35].

The psychological features which characterize BCSs at baseline were probably related to their early BC diagnosis which induced severe emotional distress and deep awareness of their own survival. At the same time BCSs showed hyperarousal during the interview, disclosing great fears of both losing their lives and not being able to control the progress of their heath. They were suffering from intense psychic and somatic anxious symptoms which impaired their perceived QoL. Particularly we found that perceived QoL in BCSs was lower at baseline as compared to controls, and it is conceivable that their oncologic pathology, even if after specific previous treatments, could lead to significant role limitations because of pain, anxiety and depressive symptoms [8,82–88].

We cannot explain this finding with a causal relationship between AIs treatment and psychological improvement, as we detected an improvement of the same psychological features in the control group too. Both groups suffered low baseline serum 25(OH)D levels and were treated by cholecalciferol supplementation at equal dosage from baseline to 6 months of observation. This vitamin D supplementation could contribute at least in part to the psychological improvement, as low levels of vitamin D in postmenopausal women are associated with depression, anxiety and low perceived QoL [47,57, 89,90], although the distance from surgical treatment could allow patients to elaborate mental processes, useful for helping them adapt better; moreover, we could consider the surgical cancer ablation made women feel free from a dramatic fear for their survival.

The awareness that their global health was at the centre of interest of both the clinical psychologist and physician probably had in part a positive influence on their emotional distress, also improving their perceived QoL. BCSs at the time of diagnosis commonly experience psychological trauma, but they could have resources to live their life with a healthier psychological approach. This could at least in part explain why they are able to benefit from other psychological sources, which could lead to useful adaptation to the stressful condition affecting them. Beyond suffering, psychological distress may also decrease the ability to find the best way to face and solve symptoms.

Furthermore, we observed that anxiety levels and perceived QoL changes in BCSs varied during the observational period in a directly proportional way. Particularly, HAM-A administration allowed us to highlight lower anxiety levels predicting a better perceived QoL, especially with regard to MCS, and independently from age, age at menopause, vitamin D status and depression.

In future research it could be valuable to plan a clinical psychological intervention strategy assisting patients to mentally integrate such chronic diseases, focusing on reducing psychological outcomes and improving QoL.

The strengths of the current study include a gold standard diagnostic interview which conferred specific objectivity to the performed surveys, and the complementary evaluation of clinical psychological features, in a homogeneous cohort of postmenopausal BCSs; the multiple regression analysis allowed us to highlight the association between Δ HAM-A and Δ MCS after multiple adjustment as depressive symptoms, age and serum 25(OH)D levels.

We must recognize that our research has some limitations as it was conducted solely in Italy, thus the findings may not be generalizable and it is based on a small sample size. Moreover, the control group consisted of postmenopausal women who had not previously suffered from BC or other malignancy, thus we could not exclude the possible effect of cancer and its treatment on the explored variables even relative to the comparison between different therapies among aromatase inhibitors. Also, adherence to AIs, side effects from AIs and participation in psychotherapy or counselling were not assessed. Further research should be conducted considering control groups of women with ER negative BC. A pain evaluation was not performed, excluding the possibility to directly apply associations between pain entity and changes in variables that could be expected in reference with PCS (e.g. for role limitation because of physical problems). Moreover, the small sample size did not allow separate sub-analysis of BCSs undergoing different AIs treatment, or even a separate analysis relative to previous therapies. Finally, the short 6- month observation period did not allow us to detect how the explored variables would change over time during these long-term prophylactic therapies.

## Conclusion

Our study showed BCSs’ higher anxious and depressive symptoms compared to controls. Our findings revealed that 6 months of AIs treatment was associated with the improvement of clinical psychological features and better health related QoL in comparison with the baseline. This data could be useful to plan BCSs psychological intervention focused on health concerns as well as for assisting patients in reducing psychological and physical consequences due to this chronic disease and its treatments.

## Supporting information

S1 Table Baseline main clinical characteristics of all participants, breast cancer survivors (BCSs) and controls. Values are expressed as mean ± SD or median (IQR) as appropriate. BMI = Body Mass Index; S-25(OH)D = 25-hydrossi-vitamin D serum level; HAM-A = Hamilton Anxiety Rating Scale; BDI-II = Beck Depression Inventory II edition; SF-36 = Short Form Survey Instrument.
(DOCX)

S2 Table Changes in psychological features in patients with breast cancer and controls at baseline and after 6 months of treatment with aromatase inhibitors. Data are reported as mean ± SD. BC = Breast Cancer; HAM-A = Hamilton Anxiety Rating Scale; BDI-II = Beck Depression Inventory II edition; PCS = Physical Component Summary; MCS = Mental Component Summary.
(DOCX)

S3 Table Correlation analysis at baseline between the studied variables of all participants. BMI = Body Mass Index; HAM-A = Hamilton Anxiety Rating Scale; BDI-II = Beck Depression Inventory II edition; PCS = Physical Component Summary; MCS = Mental Component Summary.
(DOCX)

## References

[1] Fitzmaurice C, Dicker D, Pain A, Hamavid H, Moradi-Lakeh M, MacIntyre MF, et al The global burden of cancer 2013. JAMA Oncology. 2015 7 1;1(4):505–27. 10.1001/jamaoncol.2015.0735 26181261 PMC4500822

[2] Schagen SB, Vardy J. Cognitive dysfunction in people with cancer. The Lancet Oncology. 2007 10 1;8(10):852–3. 10.1016/S1470-2045(07)70287-5 17913649

[3] Ahles TA, Root JC, Ryan EL. Cancer-and cancer treatment–associated cognitive change: an update on the state of the science. Journal of Clinical Oncology. 2012 10 20;30(30):3675 10.1200/JCO.2012.43.0116 23008308 PMC3675678

[4] Ganz PA, Petersen L, Castellon SA, Bower JE, Silverman DH, Cole SW, et al Cognitive function after the initiation of adjuvant endocrine therapy in early-stage breast cancer: an observational cohort study. Journal of Clinical Oncology. 2014 11 1;32(31):3559 10.1200/JCO.2014.56.1662 25267747 PMC4209106

[5] Harvey JM, Clark GM, Osborne CK, Allred DC. Estrogen receptor status by immunohistochemistry is superior to the ligand-binding assay for predicting response to adjuvant endocrine therapy in breast cancer. Journal of Clinical Oncology. 1999 5 1;17(5):1474–81. 10.1200/JCO.1999.17.5.1474 10334533

[6] Howell A, Cuzick J, Baum M, Buzdar A, Dowsett M, Forbes JF, et al Results of the ATAC (Arimidex, Tamoxifen, Alone or in Combination) trial after completion of 5 years’ adjuvant treatment for breast cancer. Lancet. 2005 1 1;365(9453):60–2. 10.1016/S0140-6736(04)17666-6 15639680

[7] Rocha-Cadman X, Massie MJ, Du Hamel K. Aromatase inhibitors and mood disturbances. Palliative & Supportive Care. 2012 Sep;10(3):225–7. 10.1017/S1478951512000636 PMC6048955 22677000

[8] Roberts K, Rickett K, Greer R, Woodward N. Management of aromatase inhibitor induced musculoskeletal symptoms in postmenopausal early Breast cancer: A systematic review and meta-analysis. Critical Reviews in Oncology/Hematology. 2017 3 1;111:66–80. 10.1016/j.critrevonc.2017.01.010 28259297

[9] Martino G, Catalano A, Bellone F, Russo GT, Vicario CM, Lasco A, et al As Time Goes by: Anxiety Negatively Affects the Perceived Quality of Life in Patients With Type 2 Diabetes of Long Duration. Frontiers in Psychology. 2019;10:1779 10.3389/fpsyg.2019.01779 31428028 PMC6689992

[10] Rosenberg SM, Stanton AL, Petrie KJ, Partridge AH. Symptoms and symptom attribution among women on endocrine therapy for breast cancer. The Oncologist. 2015 6 1;20(6):598–604. 10.1634/theoncologist.2015-0007 25933930 PMC4571793

[11] Marchini F, Caputo A, Napoli A, Balonan JT, Martino G, Nannini V, et al Chronic illness as loss of good self: underlying mechanisms affecting diabetes adaptation. Mediterranean Journal of Clinical Psychology. 2018 12 20;6(3). 10.6092/2282-1619/2018.6.1981

[12] Settineri S, Frisone F, Merlo EM, Geraci D, Martino G. Compliance, adherence, concordance, empowerment, and self-management: five words to manifest a relational maladjustment in diabetes. Journal of Multidisciplinary Healthcare. 2019;12:299 10.2147/JMDH.S193752 31118655 PMC6499139

[13] Martino G, Caputo A, Bellone F, Quattropani MC, Vicario C. Going Beyond the Visible in Type 2 Diabetes Mellitus: Defense Mechanisms and their Associations with Depression and Health-Related Quality of Life. Frontiers in Psychology. 2020 2; 10.3389/fpsyg.2020.00267 32174865 PMC7054284

[14] Vicario CM, Salehinejad MA, Felmingham K, Martino G, Nitsche MA. A systematic review on the therapeutic effectiveness of non-invasive brain stimulation for the treatment of anxiety disorders. Neuroscience & Biobehavioral Reviews. 2018 12 10 10.1016/j.neubiorev.2018.12.012 30543906

[15] Martino G, Bellone F, Langher V, Caputo A, Catalano A, Quattropani MC, et al Alexithymia and Psychological Distress Affect Perceived Quality of Life in Patients with Type 2 Diabetes Mellitus. Mediterranean Journal of Clinical Psychology. 2019 12 12;7(3).

[16] Martino G, Langher V, Cazzato V, Vicario CM. Psychological factors as determinants of medical conditions. Frontiers in psychology. 2019;10:2502 10.3389/fpsyg.2019.02502 31781002 PMC6857525

[17] Lenzo V, Sardella A, Martino G, Quattropani MC. A systematic review of metacognitive beliefs in chronic medical conditions. Front. Psychol. 10: 2875 10.3389/fpsyg.2019.02875 2020 1 10 31998178 PMC6965316

[18] Dell’Osso L, Stratta P, Conversano C, Massimetti E, Akiskal KK, Akiskal HS, et al Lifetime mania is related to post-traumatic stress symptoms in high school students exposed to the 2009 L’Aquila earthquake. Comprehensive Psychiatry. 2014 2 1;55(2):357–62. 10.1016/j.comppsych.2013.08.017 24269194

[19] Dell'Osso L, Carmassi C, Rucci P, Ciapparelli A, Conversano C, Marazziti D. Complicated grief and suicidality: the impact of subthreshold mood symptoms. CNS Spectrums. 2011 Jan;16(1):1–6. 10.1017/S1092852912000090 24725296

[20] Carmassi C, Shear MK, Massimetti G, Wall M, Mauro C, Gemignani S, et al Validation of the Italian version Inventory of Complicated Grief (ICG): A study comparing CG patients versus bipolar disorder, PTSD and healthy controls. Comprehensive Psychiatry. 2014 7 1;55(5):1322–9. 10.1016/j.comppsych.2014.03.001 24721191

[21] Piccinni A, Origlia N, Veltri A, Vizzaccaro C, Marazziti D, Catena-Dell'Osso M, et al Plasma β-amyloid peptides levels: a pilot study in bipolar depressed patients. Journal of Affective Disorders. 2012 4 1;138(1–2):160–4. 10.1016/j.jad.2011.12.042 22310032

[22] Martino G, Sardella A, Bellone F, Lasco C, Langher V, Cazzato V, et al Executive functions and bone health: a focus on cognitive impulsivity and bone mineral density. Mediterranean Journal of Clinical Psychology. 2019 8 1;7(2). 10.6092/2282-1619/2019.7.2167

[23] Catalano A, Sardella A, Bellone F, Lasco CG, Martino G, Morabito N. Executive functions predict fracture risk in postmenopausal women assessed for osteoporosis. Aging clinical and experimental research. 2019 Nov 26:1–7. 10.1007/s40520-019-01426-w 31773488

[24] Catalano A, Martino G, Bellone F, Papalia M, Lasco C, Basile G, et al Neuropsychological Assessment in Elderly Men with Benign Prostatic Hyperplasia Treated with Dutasteride. Clinical drug investigation. 2019 1 31;39(1):97–102. 10.1007/s40261-018-0720-7 30367429

[25] Barberis N, Quattropani MC, Cuzzocrea F. Relationship between motivation, adherence to diet, anxiety symptoms, depression symptoms and quality of life in individuals with celiac disease. Journal of psychosomatic research. 2019 9 1;124:109787 10.1016/j.jpsychores.2019.109787 31443802

[26] Marchi L, Marzetti F, Orrù G, Lemmetti S, Miccoli M, Ciacchini R, et al Alexithymia and psychological distress in patients with fibromyalgia and rheumatic disease. Frontiers in psychology. 2019;10 10.3389/fpsyg.2019.00010 31417462 PMC6685004

[27] Sheikh S, Dahiya S, Ansari AH, Kumar MM. The association of quality of life between anxiety and depression in patients with chronic rheumatic heart disease. Mediterranean Journal of Clinical Psychology. 2019 8 10;7(2). 10.6092/2282-1619/2019.7.2164

[28] Castelnuovo G, Pietrabissa G, Manzoni GM, Corti S, Ceccarini M, Borrello M, et al Chronic care management of globesity: promoting healthier lifestyles in traditional and mHealth based settings. Frontiers in Psychology. 2015 10 15;6:1557 10.3389/fpsyg.2015.01557 26528215 PMC4606044

[29] Rahnea Nița RA., Popescu M., Ciuhu AN., et al The relationship between anxiety, depression and sense of illness understanding in palliative cancer patients. Archives of the Balkan Medical Union. 2016 51, 25–28.

[30] Rahnea-Nita RA, Paunica S, Motofei C, Rahnea-Nita G. Assessment of anxiety and depression in patients with advanced gynaecological cancer. Mediterranean Journal of Clinical Psychology. 2019 8 10;7(2). 10.6092/2282-1619/2019.7.2214

[31] Hermelink K, Henschel V, Untch M, Bauerfeind I, Lux MP, Munzel K. Short‐term effects of treatment‐induced hormonal changes on cognitive function in breast cancer patients: results of a multicenter, prospective, longitudinal study. Cancer. 2008 11 1;113(9):2431–9. 10.1002/cncr.23853 18823033

[32] Ribi K, Aldridge J, Phillips KA, Thompson A, Harvey V, Thürlimann B, et al Subjective cognitive complaints one year after ceasing adjuvant endocrine treatment for early-stage breast cancer. British Journal of Cancer. 2012 5;106(10):1618–25. 10.1038/bjc.2012.156 22531635 PMC3349183

[33] Frank JS, Vance DE, Jukkala A, Meneses KM. Attention and memory deficits in breast cancer survivors: implications for nursing practice and research. Journal of Neuroscience Nursing. 2014 10 1;46(5):274–84. 10.1097/JNN.0000000000000078 25099062

[34] Ates O, Soylu C, Babacan T, Sarici F, Kertmen N, Allen D, et al Assessment of psychosocial factors and distress in women having adjuvant endocrine therapy for breast cancer: the relationship among emotional distress and patient and treatment-related factors. SpringerPlus. 2016 12 1;5(1):486. 27218001 10.1186/s40064-016-2136-2 PMC4837751

[35] Maass SW, Roorda C, Berendsen AJ, Verhaak PF, de Bock GH. The prevalence of long-term symptoms of depression and anxiety after breast cancer treatment: a systematic review. Maturitas. 2015 9 1;82(1):100 10.1016/j.maturitas.2015.04.010 25998574

[36] Bidstrup PE, Christensen J, Mertz BG, Rottmann N, Dalton SO, Johansen C. Trajectories of distress, anxiety, and depression among women with breast cancer: Looking beyond the mean. Acta Oncol. 2015 5;54(5):789–96. 10.3109/0284186X.2014.1002571 25761086

[37] Vance V, Mourtzakis M, Hanning R. Relationships Between Weight Change and Physical and Psychological Distress in Early-Stage Breast Cancer Survivors. Cancer Nurs. 2019 May-Jun;42(3):E43–E50. 10.1097/NCC.0000000000000612 29847347

[38] Henselmans I, Helgeson VS, Seltman H, de Vries J, Sanderman R, Ranchor AV. Identification and prediction of distress trajectories in the first year after a breast cancer diagnosis. Health Psychol. 2010 3;29(2):160–8. 10.1037/a0017806 20230089

[39] Kant J, Czisch A, Schott S, Siewerdt-Werner D, Birkenfeld F, Keller M. Identifying and predicting distinct distress trajectories following a breast cancer diagnosis—from treatment into early survival. J Psychosom Res. 2018 12;115:6–13. 10.1016/j.jpsychores.2018.09.012 30470319

[40] Junghaenel DU, Cohen J, Schneider S, Neerukonda AR, Broderick JE. Identification of distinct fatigue trajectories in patients with breast cancer undergoing adjuvant chemotherapy. Support Care Cancer. 2015 9;23(9):2579–87. 10.1007/s00520-015-2616-x 25876159 PMC4516710

[41] Lam WW, Soong I, Yau TK, Wong KY, Tsang J, Yeo W, et al The evolution of psychological distress trajectories in women diagnosed with advanced breast cancer: a longitudinal study. Psychooncology. 2013 12;22(12):2831–9. 10.1002/pon.3361 24038545

[42] Stanton AL. What happens now? Psychosocial care for cancer survivors after medical treatment completion. J Clin Oncol 2012;30:1215–1220. 10.1200/JCO.2011.39.7406 22412133

[43] Ho SS, So WK, Leung DY, Lai ET, Chan CW. Anxiety, depression and quality of life in Chinese women with breast cancer during and after treatment: a comparative evaluation. Eur J Oncol Nurs. 2013 12;17(6):877–82. 10.1016/j.ejon.2013.04.005 23727448

[44] Takei H, Ohsumi S, Shimozuma K, Takehara M, Suemasu K, Ohashi Y, et al Health-related quality of life, psychological distress, and adverse events in postmenopausal women with breast cancer who receive tamoxifen, exemestane, or anastrozole as adjuvant endocrine therapy: National Surgical Adjuvant Study of Breast Cancer 04 (N-SAS BC 04). Breast Cancer Res Treat. 2012 5;133(1):227–36. 10.1007/s10549-011-1943-y 22234519

[45] Donovan KA, Gonzalez BD, Small BJ, Andrykowski MA, Jacobsen PB. Depressive symptom trajectories during and after adjuvant treatment for breast cancer. Ann Behav Med. 2014 6;47(3):292–302. 10.1007/s12160-013-9550-2 24158626 PMC4313122

[46] Schilder CM, Eggens PC, Seynaeve C, Linn SC, Boogerd W, Gundy CM, et al Neuropsychological functioning in postmenopausal breast cancer patients treated with tamoxifen or exemestane after AC-chemotherapy: cross-sectional findings from the neuropsychological TEAM-side study. Acta Oncologica. 2009 1 1;48(1):76–85. 10.1080/02841860802314738 18777410

[47] Catalano A, Martino G, Bellone F, Gaudio A, Lasco C, Langher V, et al Anxiety levels predict fracture risk in postmenopausal women assessed for osteoporosis. Menopause. 2018 10 1;25(10):1110–5. 10.1097/GME.0000000000001123 29738418

[48] American Psychiatric Association. Diagnostic and Statistical Manual of Mental Disorders, 5th ed Washington, DC: American Psychiatric Association; 2013.

[49] Rickham PP. Human experimentation. Code of ethics of the world medical association. Declaration of Helsinki. British Medical Journal. 1964 7;2(5402):177 10.1136/bmj.2.5402.177 14150898 PMC1816102

[50] Fava GA, Tomba E, Sonino N. Clinimetrics: the science of clinical measurements. International Journal of Clinical Practice. 2012 1;66(1):11–5. 10.1111/j.1742-1241.2011.02825.x 22171900

[51] Conti C, Carrozzino D, Patierno C, Vitacolonna E, Fulcheri M. The clinical link between type D personality and diabetes. Frontiers in Psychiatry. 2016 6 21;7:113 10.3389/fpsyt.2016.00113 27445869 PMC4914509

[52] Langher V, Caputo A, Martino G. What happened to the clinical approach to case study in psychological research? A clinical psychological analysis of scientific articles in high impact-factor journals. Mediterranean Journal of Clinical Psychology. 2017 12 30;5(3).

[53] Beck AT, Steer RA, Brown GK. Beck Depression Inventory-II. San Antonio. 1996;78(2):490–8.

[54] Hamilton MA. The assessment of anxiety states by rating. British Journal of Medical Psychology. 1959 3;32(1):50–5. 10.1111/j.2044-8341.1959.tb00467.x 13638508

[55] Ware JE Jr, Sherbourne CD. The MOS 36-item short-form health survey (SF-36): I. Conceptual framework and item selection. Medical Care. 1992 6 1:473–83. 10.1097/00005650-199206000-00002 1593914

[56] Apolone G, Mosconi P. The Italian SF-36 Health Survey: translation, validation and norming. Journal of Clinical Epidemiology. 1998 11 1;51(11):1025–36. 10.1016/s0895-4356(98)00094-8 9817120

[57] Martino G, Catalano A, Bellone F, Sardella A, Lasco C, Caprì T, et al Vitamin D status is associated with anxiety levels in postmenopausal women evaluated for osteoporosis. Mediterranean Journal of Clinical Psychology. 2018 4 27;6(1).

[58] Catalano A, Gaudio A, Morabito N, Basile G, Agostino RM, Xourafa A, et al Quantitative ultrasound and DXA measurements in aromatase inhibitor-treated breast cancer women receiving denosumab. Journal of Endocrinological Investigation. 2017 8 1;40(8):851–7. 10.1007/s40618-016-0606-6 28332172

[59] Deleskog L, Laursen NØ, Nielsen BR, Schwarz P. Vertebral fracture assessment by DXA is inferior to X-ray in clinical severe osteoporosis. Osteoporosis International. 2016 7 1;27(7):2317–26. 10.1007/s00198-016-3532-8 26892040

[60] Suppli NP, Johansen C, Christensen J, Kessing LV, Kroman N, Dalton SO. Increased risk for depression after breast cancer: a nationwide population-based cohort study of associated factors in Denmark, 1998–2011. J Clin Oncol 2014;32:3831–3839. 10.1200/JCO.2013.54.0419 25349294

[61] Nabholtz JM, Buzdar A, Pollak M, Harwin W, Burton G, Mangalik A, et al Anastrozole is superior to tamoxifen as first-line therapy for advanced breast cancer in postmenopausal women: results of a North American multicenter randomized trial. Journal of Clinical Oncology. 2000 11 15;18(22):3758–67. 10.1200/JCO.2000.18.22.3758 11078488

[62] Coates AS, Keshaviah A, Thurlimann B, Mouridsen H, Mauriac L, Forbes JF, et al Five years of letrozole compared with tamoxifen as initial adjuvant therapy for postmenopausal women with endocrine-responsive early breast cancer: update of study BIG 1–98. Journal of Clinical Oncology. 2007 2 10;25(5):486–92. 10.1200/JCO.2006.08.8617 17200148

[63] Coombes RC, Hall E, Gibson LJ, Paridaens R, Jassem J, Delozier T, et al A randomized trial of exemestane after two to three years of tamoxifen therapy in postmenopausal women with primary breast cancer. New England Journal of Medicine. 2004 3 11;350(11):1081–92. 10.1056/NEJMoa040331 15014181

[64] Norbury R, Cutter WJ, Compton J, Robertson DM, Craig M, Whitehead M, et al The neuroprotective effects of estrogen on the aging brain. Experimental Gerontology. 2003 1 1;38(1–2):109–17. 10.1016/s0531-5565(02)00166-3 12543268

[65] Cholerton B, Gleason CE, Baker LD, Asthana S. Estrogen and Alzheimer’s disease. Drugs & Aging. 2002 6 1;19(6):405–27. 12149049 10.2165/00002512-200219060-00002

[66] Cheng CM, Cohen M, Wang JI, Bondy CA. Estrogen augments glucose transporter and IGF1 expression in primate cerebral cortex. The FASEB Journal. 2001 4;15(6):907–15. 10.1096/fj.00-0398com 11292650

[67] Rossouw JE. Writing Group for the Women's Health Initiative Investigators. Risks and benefits of estrogen plus progestin in healthy postmenopausal women: principal results from the Women's Health Initiative randomized controlled trial. Jama. 2002;288:321–33. 10.1001/jama.288.3.321 12117397

[68] Shumaker SA. Women's Health Initiative Memory Study. Conjugated equine estrogens and incidence of probable dementia and mild cognitive impairment in postmenopausal women: Women's Health Initiative Memory Study. JAMA. 2004;291:2947–58. 10.1001/jama.291.24.2947 15213206

[69] Hurria A, Patel SK, Mortimer J, Luu T, Somlo G, Katheria V, et al The effect of aromatase inhibition on the cognitive function of older patients with breast cancer. Clinical Breast Cancer. 2014 4 1;14(2):132–40. 10.1016/j.clbc.2013.10.010 24291380 PMC4103787

[70] Matthews K, Cauley J, Yaffe K, Zmuda JM. Estrogen replacement therapy and cognitive decline in older community women. Journal of the American Geriatrics Society. 1999 5;47(5):518–23. 10.1111/j.1532-5415.1999.tb02563.x 10323642

[71] Hara Y, Waters EM, McEwen BS, Morrison JH. Estrogen effects on cognitive and synaptic health over the lifecourse. Physiological Reviews. 2015 6 24;95(3):785–807. 10.1152/physrev.00036.2014 26109339 PMC4491541

[72] Bender CM, Sereika SM, Ryan CM, Brufsky AM, Puhalla S, Berga SL. Does lifetime exposure to hormones predict pretreatment cognitive function in women before adjuvant therapy for breast cancer? Menopause. 2013 9;20(9):922–9. 10.1097/GME.0b013e3182843eff 23481123 PMC3745534

[73] Bender CM, Merriman JD, Gentry AL, Ahrendt GM, Berga SL, Brufsky AM, et al Patterns of change in cognitive function with anastrozole therapy. Cancer. 2015 8 1;121(15):2627–36. 10.1002/cncr.29393 25906766 PMC4512875

[74] Merriman JD, Sereika SM, Brufsky AM, McAuliffe PF, McGuire KP, Myers JS, et al Trajectories of self-reported cognitive function in postmenopausal women during adjuvant systemic therapy for breast cancer. Psychooncology. 2017 1;26(1):44–52. 10.1002/pon.4009 26486371 PMC4969219

[75] Bender CM, Merriman JD, Sereika SM, Gentry AL, Casillo FE, Koleck TA, et al Trajectories of Cognitive Function and Associated Phenotypic and Genotypic Factors in Breast Cancer. Oncol Nurs Forum. 2018 5 1;45(3):308–326. 10.1188/18.ONF.308-326 29683114 PMC6052441

[76] Bender CM, Sereika SM, Brufsky AM, Ryan CM, Vogel VG, Rastogi P, et al Memory impairments with adjuvant anastrozole versus tamoxifen in women with early-stage breast cancer. Menopause. 2007 Nov-Dec;14(6):995–8. 10.1097/gme.0b013e318148b28b 17898668 PMC2831410

[77] Bender CM, Merriman JD. Cancer- and treatment-related cognitive changes: what can we do now? What lies ahead? Oncology (Williston Park). 2014 9;28(9):806–8. 25224482 PMC4261918

[78] Taira N, Iwata H, Hasegawa Y, Sakai T, Higaki K, Kihara K, et al Health-related quality of life and psychological distress during neoadjuvant endocrine therapy with letrozole to determine endocrine responsiveness in postmenopausal breast cancer. Breast Cancer Research and Treatment. 2014 5 1;145(1):155–64. 10.1007/s10549-014-2935-5 24692082

[79] Zigmond AS, Snaith RP. The hospital anxiety and depression scale. Acta Psychiatrica Scandinavica. 1983 6;67(6):361–70. 10.1111/j.1600-0447.1983.tb09716.x 6880820

[80] Vodermaier A, Millman RD. Accuracy of the Hospital Anxiety and Depression Scale as a screening tool in cancer patients: a systematic review and meta-analysis. Supportive Care in Cancer. 2011 12 1;19(12):1899 10.1007/s00520-011-1251-4 21898134

[81] Norton S, Cosco T, Doyle F, Done J, Sacker A. The Hospital Anxiety and Depression Scale: a meta confirmatory factor analysis. Journal of Psychosomatic Research. 2013 1 1;74(1):74–81. 10.1016/j.jpsychores.2012.10.010 23272992

[82] Watt T, Hegedüs L, Bjorner JB, Groenvold M, Bonnema SJ, Rasmussen ÅK, et al Is thyroid autoimmunity per se a determinant of quality of life in patients with autoimmune hypothyroidism?. European Thyroid Journal. 2012;1(3):186–92. 10.1159/000342623 24783018 PMC3821477

[83] Bové KB, Watt T, Vogel A, Hegedüs L, Bjoerner JB, Groenvold M, et al Anxiety and depression are more prevalent in patients with graves' disease than in patients with nodular goitre. European Thyroid Journal. 2014;3(3):173–8. 10.1159/000365211 25538899 PMC4224229

[84] Catalano A, Martino G, Morabito N, Scarcella C, Gaudio A, Basile G, et al Pain in osteoporosis: from pathophysiology to therapeutic approach. Drugs & Aging. 2017 10 1;34(10):755–65. 28980156 10.1007/s40266-017-0492-4

[85] Lauriola M, Tomai M, Palma R, La Spina G, Foglia A, Panetta C, et al Intolerance of uncertainty and anxiety-related dispositions predict pain during upper endoscopy. Frontiers in Psychology. 2019;10:1112 10.3389/fpsyg.2019.01112 31156518 PMC6529782

[86] Fiegl S, Lahmann C, O'Rourke T, Probst T, Pieh C. Depression according to ICD-10 clinical interview vs. depression according to the Epidemiologic Studies Depression Scale to predict pain therapy outcomes. Frontiers in Psychology. 2019;10:1862 10.3389/fpsyg.2019.01862 31481912 PMC6711408

[87] Di Giuseppe M, Ciacchini R, Micheloni T, Bertolucci I, Marchi L, Conversano C. Defense mechanisms in cancer patients: A systematic review. Journal of psychosomatic research. 2018 12 1;115:76–86. 10.1016/j.jpsychores.2018.10.016 30470322

[88] Di Giuseppe M, Ciacchini R, Piarulli A, Nepa G, Conversano C. Mindfulness dispositions and defense style as positive responses to psychological distress in oncology professionals. European Journal of Oncology Nursing. 2019 6 1;40:104–10. 10.1016/j.ejon.2019.04.003 31229199

[89] Martino G, Catalano A, Bellone F, Langher V, Lasco C, Penna A, et al Quality of life in postmenopausal women: which role for vitamin D?. Mediterranean Journal of Clinical Psychology. 2018 8 21;6(2).

[90] Husemoen LL, Ebstrup JF, Mortensen EL, Schwarz P, Skaaby T, Thuesen BH, et al Serum 25-hydroxyvitamin D and self-reported mental health status in adult Danes. European Journal of Clinical Nutrition. 2016 1;70(1):78 10.1038/ejcn.2015.129 26264349

