# Supplementary material for: Quality of life and psychological functioning in postmenopausal women undergoing aromatase inhibitor treatment for early breast cancer
Source: PLoS One. 2020 Mar 26;15(3):e0230681. doi: 10.1371/journal.pone.0230681 (PMC7098625; doi:10.1371/journal.pone.0230681)
Supplement: S1 Table — Values are expressed as mean ± SD or median (IQR) as appropriate. BMI = Body Mass Index; S-25(OH)D = 25-hydrossi-vitamin D serum level; HAM-A = Hamilton Anxiety Rating Scale; BDI-II = Beck Depression Inventory II edition; SF-36 = Short Form Survey Instrument. (DOCX) [file pone.0230681.s001.docx]

|  | **Total**  *(n=102)* | **BCSs**  *(n=51)* | **Controls**  *(n=51)* | **p value** |
| --- | --- | --- | --- | --- |
| **Age** *(yr.)* | 66.5±9.1 | 66.9±8.7 | 66±10.9 | NS |
| **Age at menopause** *(yr.)* | 47.3±5.4 | 47.±4.8 | 47.5±5.1 | NS |
| **BMI** *(Kg/m^2^)* | 24.4±4.9 | 24.5±5.5 | 24.4±4.2 | NS |
| **Current smoking** *[n(%)]* | 10 (10) | 5 (10) | 5 (10) | NS |
| **Alcohol** **≥ 3units/day** *[n(%)]* | 0 | 0 | 0 | NS |
| **S-25(OH)D** *(ng/ml)* | 26.9±10 | 25.1±7.4 | 28.7±11.7 | NS |
|  |  |  |  |  |
| **Bone mineral density** |  |  |  |  |
| **Lumbar spine T-score** *(SD)* | -2 ± 1 | -2.1 ± 1 | -1.9 ± 0.9 | NS |
| **Femoral neck T-score** *(SD)* | -1.8 ± 0.6 | -1.8 ± 0.6 | -1.8 ± 0.7 | NS |
|  |  |  |  |  |
| **Anxiety levels** |  |  |  |  |
| **HAM-A score** | 27.8±7.11 | 33.2±4.1 | 22.3±5^*^ | <0.0001 |
| **HAM-A somatic symptom score** | 11.9±3.9 | 14.5±2.8 | 9.3±3.2 | <0.0001 |
| **HAM-A psychic symptom score** | 15.9±3.8 | 18.7±2.4 | 13±2.9 | <0.0001 |
|  |  |  |  |  |
| **Depression severity** |  |  |  |  |
| **BDI-II score** | 7.2±3.1 | 8.6±2.6 | 5.9±3.1 | <0.0001 |
|  |  |  |  |  |
| **Perceived Quality of Life – SF-36** |  |  |  |  |
| **Mental health** | 28 (20 to 52) | 28 (17 to 32) | 44 (21 to 56) | 0.001 |
| **Role emotional** | 0 (0 to 33) | 0 (0 to 0) | 33 (0 to 66) | <0.0001 |
| **Social functioning** | 50 (25 to 62) | 37 (25 to 50) | 50 (37 to 62) | 0.0003 |
| **Vitality** | 35 (25 to 50) | 30 (20 to 40) | 40 (30 to 55) | 0.0008 |
| **General health** | 40 (25 to 52) | 30 (20 to 40) | 45 (35 to 52) | 0.0011 |
| **Bodily pain** | 41 (22 to 52) | 30 (22 to 41) | 41 (22 to 74) | 0.004 |
| **Role physical** | 0 (0 to 50) | 0 (0 to 0) | 25 (0 to 75) | 0.0001 |
| **Physical functioning** | 55 (30 to 75) | 35 (20 to 55) | 75 (55 to 90) | <0.0001 |

**S1 Table**. **Baseline main clinical characteristics of all participants, breast cancer survivors (BCSs) and controls.**

*Values are expressed as mean ± SD or median (IQR) as appropriate. BMI = Body Mass Index; S-25(OH)D = 25-hydrossi-vitamin D serum level; HAM-A = Hamilton Anxiety Rating Scale; BDI-II = Beck Depression Inventory II edition; SF-36 = Short Form Survey Instrument. Data are reported as mean ± SD or median (IQR) as appropriate. BCSs = breast cancer survivors; HAM-A = Hamilton Anxiety; BDI-II = Beck Depression Inventory II edition.*
