# Supplementary material for: Quality of life and psychological functioning in postmenopausal women undergoing aromatase inhibitor treatment for early breast cancer
Source: PLoS One. 2020 Mar 26;15(3):e0230681. doi: 10.1371/journal.pone.0230681 (PMC7098625; doi:10.1371/journal.pone.0230681)
Supplement: S2 Table — Data are reported as mean ± SD. BC = Breast Cancer; HAM-A = Hamilton Anxiety Rating Scale; BDI-II = Beck Depression Inventory II edition; PCS = Physical Component Summary; MCS = Mental Component Summary. (DOCX) [file pone.0230681.s002.docx]

**S2 Table**. **Changes in psychological features in patients with breast cancer and controls at baseline and after 6 months of treatment with aromatase inhibitors.**

|  | **BC**  *(n=51)* | |  | **Controls**  *(n=51)* | |  |
| --- | --- | --- | --- | --- | --- | --- |
|  | Baseline | 6 months |  | Baseline | 6 months |  |
| **Anxiety levels** |  |  | p value |  |  | p value |
| HAM-A score | 33.2±4.1 | 30±3.7 | <0.001 | 22.3±5 | 20±4.4 | <0.001 |
| HAM-A somatic symptom score | 14.5±2.8 | 13.2±2.6 | 0.02 | 9.3±3.2 | 8.2±2.5 | 0.05 |
| HAM-A psychic symptom score | 18.7±2.4 | 17.2±2.2 | 0.002 | 13±2.9 | 11.9±2.8 | 0.04 |
| **Depression severity** |  |  |  |  |  |  |
| BDI-II score | 8.6±2.6 | 8±2.6 | 0.001 | 5.9±3 | 5.6±2 | NS |
| **Perceived quality of life** |  |  |  |  |  |  |
| PCS | 32.3±7.2 | 33.8±6.1 | 0.006 | 40.5±9.1 | 45.4±8 | <0.001 |
| MCS | 27.9±5.4 | 34±6.2 | <0.001 | 33.5±9.8 | 39.3±9.2 | <0.001 |

*BC = Breast Cancer; HAM-A = Hamilton Anxiety* *Rating Scale; BDI-II = Beck Depression Inventory II edition; PCS = Physical Component Summary; MCS = Mental Component Summary. Data are reported as mean ± SD.*
