# Supplementary material for: Quality of life and psychological functioning in postmenopausal women undergoing aromatase inhibitor treatment for early breast cancer
Source: PLoS One. 2020 Mar 26;15(3):e0230681. doi: 10.1371/journal.pone.0230681 (PMC7098625; doi:10.1371/journal.pone.0230681)
Supplement: S3 Table — BMI = Body Mass Index; HAM-A = Hamilton Anxiety Rating Scale; BDI-II = Beck Depression Inventory II edition; PCS = Physical Component Summary; MCS = Mental Component Summary. (DOCX) [file pone.0230681.s003.docx]

**S3 Table.**  **Correlation analysis at baseline between the studied variables of all participants.**

|  | **Age** | **Age at menopause** | **BMI** | **HAM-A score** | **BDI-II score** | **PCS** | **MCS** |
| --- | --- | --- | --- | --- | --- | --- | --- |
| **Age** |  | r=0.13; p=0.19 | r=0.1; p=0.28 | r=0.15; p=0.12 | **r=0.25; p=0.01** | **r=-0.52; p<0.001** | **r=-0.34; p<0.001** |
| **Age at menopause** | r=0.13; p=0.19 |  | r=0.11; p=0.27 | r=-0.03; p=0.74 | r=0.07; p=0.49 | r=-0.14; p=0.16 | r=-0.14; p=0.16 |
| **BMI** | r=0.1; p=0.28 | r=0.11; p=0.27 |  | r=-0.12; p=0.21 | r=-0.15; p=0.12 | r=-0.08; p=0.38 | r=0.10; p=0.31 |
| **HAM-A score** | r=0.15; p=0.12 | r=-0.03; p=0.74 | r=-0.12; p=0.21 |  | **r=0.63; p<0.001** | **r=-0.49; p<0.001** | **r=-0.53; p<0.001** |
| **BDI-II score** | **r=0.25; p=0.01** | r=0.07; p=0.49 | r=-0.15; p=0.12 | **r=0.63; p<0.001** |  | **r=-0.54; p<0.001** | **r=-0.46; p<0.001** |
| **PCS** | **r=-0.52; p<0.001** | r=-0.14; p=0.16 | r=-0.08; p=0.38 | **r=-0.49; p<0.001** | **r=-0.54; p<0.001** |  | **r=0.39; p<0.001** |
| **MCS** | **r=-0.34; p<0.001** | r=-0.14; p=0.16 | r=0.10; p=0.31 | **r=-0.53; p<0.001** | **r=-0.46; p<0.001** | **r=0.39; p<0.001** |  |

*BMI = Body Mass Index; HAM-A = Hamilton Anxiety* *Rating Scale; BDI-II = Beck Depression Inventory II edition; PCS = Physical Component Summary; MCS = Mental Component Summary.*
